# Supplementary material for: Encapsulation of catalyst in block copolymer micelles for the polymerization of ethylene in aqueous medium
Source: Nat Commun. 2018 Feb 26;9:841. doi: 10.1038/s41467-018-03253-5 (PMC5827096; doi:10.1038/s41467-018-03253-5)
Supplement: Supplementary file 1 — Supplementary Information [file 41467_2018_3253_MOESM1_ESM.pdf]

SUPPLEMENTARY INFORMATION

Ethylene Polymerization in Aqueous Medium via Encapsulation of Catalyst in Block Copolymer Micelles

Damien Guironnet et al.

Supplementary Methods

Materials.

All manipulations of metal complexes were carried out under an inert atmosphere using glove box or Schlenk techniques. Solvents were dried and degassed. Deionized water was degassed with argon before use. Unless otherwise stated reagents obtained from the commercial supplier were used without any purification. Ligand L_1 was obtained from Sigma Aldrich and L_2 was synthesized as reported in literature method.[1]

Instrumentation.

Gel Permeation Chromatography was performed using a Tosoh Ecosec HLC-8320GPC fitted with a reference column (6.0 mm ID x 15 cm) a guard column (6.0 mm ID x 4.0 cm x 5 µm) and two analytical columns (7.8 mm ID x 30 cm x 5 µm). Mobile phase is THF HPLC grade where the reference flow rate is 0.5 ml/min while the analytical column is at 1.0 $ml.min^{-1}$. Samples were dissolved in THF HPLC grade (5-10 $mg.ml^{-1}$) and filtered through a 0.45 µm PTFE syringe filter. Molecular weights were determined against polystyrene standards (15 points ranging from 500 Mw to 8.42 million Mw). High-temperature Gel permeation chromatography (GPC) was performed at 160 °C in 1,2,4-trichlorobenzene at a flow rate of 1.0 $mL.min^{-1}$ on a Malvern Viscotek HT-GPC equipped with triple detection. Transmission electron microscopy images were obtained using JEOL 2100 Cryo microscope. High temperature NMR were acquired on the UI600 NMR a VARIAN UNITY INOVA 600 NB equipped with a 5mm Varian AutoTuneX $^1H/X$ PFG Z probe. Particles size distribution were measure by DLS on a Malvern Zetasizer ZS90.

BCP Synthesis.

PEG-Br Macroinitiator

Poly(ethylene glycol) methyl ether (PEG, Mn = 5000 g mol^{-1}, 22.6 g, 4.5 mmol) was dissolved in dichloromethane (150 mL). Triethylamine (2.5 mL, 18 mmol, 4.0 equiv.) was added at 0°C, followed by the slow addition of 2-bromoisobutyryl bromide (1.7 mL, 13.5 mmol, 3 equiv.) at the same temperature. The reaction mixture was brought to room temperature and stirred for 24 h. The crude product was filtered through a short plug of silica. The filtrate was concentrated under reduced pressure. The product was dissolved in tetrahydrofuran (10 mL) and precipitated in cold diethyl ether (200 mL) and isolated as a white powder. The precipitation step was repeated three times. The polymer was dried under vacuum for 24 h at 50°C. (20.4 g, 88% yield)

^{1}H NMR (in $CDCl_3$, 500 MHz): δ 4.3 (br. s, CO_2CH_2), 3.6 (br. m, OCH_2CH_2, 456H), 3.3 (s, OCH_3, 3H), 1.9 (s, 6H, CH_3). ^{13}C NMR (in $CDCl_3$, 500 MHz): δ 171.6, 71.9, 70.7, 68.7, 65.1, 59.0, 55.7, 45.9, 30.7, 8.6, Mn (GPC) = 3100, Đ = 1.1.

PEG-b-PCF$_3$ Block Copolymer

(The same procedure was used to synthesize all the BCP used)

In a flame dry Schlenk flask, PEG–Br (5.0 g, Mn = 5200 g mol^{-1}, 0.96 mmol), trifluoroethyl methacrylate (5.6 g, 33.6 mmol, 35 equiv.), bipyridine (351 mg, 2.2 mmol, 2.3 equiv.), CuBr (141.0 mg, 0.96 mmol), and $CuBr_2$ (20 mg, 0.089 mmol) were combined and dissolved in trifluoro toluene (10 mL). The mixture was degassed via freeze–pump–thaw for 4-cycles. The reaction flask was placed in a pre-heated oil bath at 75°C. After 2.5 h, the polymerization was stopped, the flask was immediately cooled and exposed to air. The crude polymer was filtered thru a short plug of silica (bottom layer) and alumina neutral (top layer). The column was rinsed with excess tetrahydrofuran. The crude solution was concentrated under reduced pressure.

Tetrahydrofuran (10 mL) was added to dissolve the crude polymer and poured into cold diethyl ether (100 mL). The slurry was separated by centrifugation at 5000 rpm. The polymer was dried under vacuum for 24 h at 50°C. (4.1 g, 42% yield)

^{1}H NMR (in $CDCl_3$ at 500 MHz): δ 4.3 (br. s, $CO_2CH_2CF_3$, 54H), 3.6 (br. m, OCH_2CH_2, 456H), 2.0-1.8 (br, 54H, main chain CH_2), 1.0-0.8 (br, 81H, acrylate CH_3). ^{13}C (in $CDCl_3$ at 500 MHz): δ 175.2, 122.8, 71.9, 70.7, 61.2, 59.0, 45.5, 30.7, 18.6, 16.7

PCF_3 homopolymer

PCF_3 homopolymer was synthesized following the same procedure as PEG-*b*-PCF_3 using ethyl α-bromoisobutyrate as macro initiator.

PEG-OCOMe homopolymer

In a flame dry flask, PEG–OH (5.0 g, Mn=5000, 1.0 mmol) was dissolved in dichloromethane (50 mL). Pyridine (0.4 mL, 5 mmol, 5.0 equiv.), DMAP (61 mg, 0.5 mmol, 0.5 equiv.) and acetic anhydride (0.31 mL, 5 mmol, 5 equiv.) were added at 0°C. The reaction mixture was brought to room temperature and stirred overnight. The product was obtained with the same work up as in the synthesis of PEG-Br.

Supplementary Table 1 Characteristics of the BCP

BCP	M_n[a]	M_n[b]	Đ[b]	Number of repeat units
PEG$_n$-*b*-PEHA$_m$	9970	9330	1.2	n=114<br>m=27
PEG$_n$-*b*-PCF$_{3m}$	9500	5700	1.2	n=114<br>m=27
PEG$_n$-*b*-PS$_m$	8500	7800	1.1	n=114<br>m=34

a) Determined by NMR analysis using the ratio of methyl ether proton versus the repeating methylene proton of the repeating units, b) GPC analysis (Mn determined versus PS standard), c) Estimate number of units using the ratio of methyl ether proton versus methyl end group

Homopolymers

PEG-OH **PCF$_3$** **PEG-OCOMe**

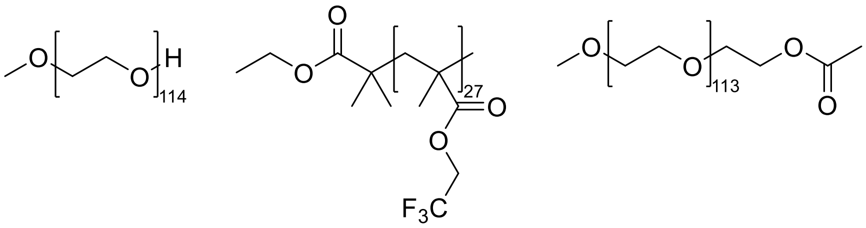

Catalyst Synthesis

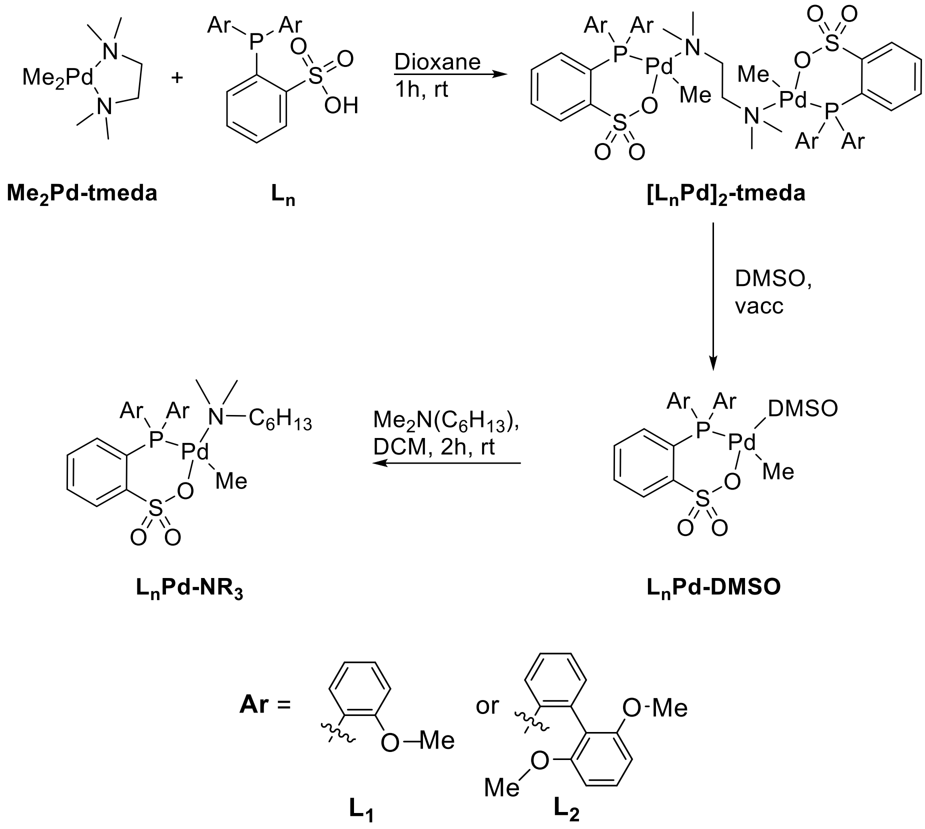

Supplementary Figure 1 Synthetic routes of **L_1Pd-NR$_3$** and **L_2Pd-DMSO**

Synthesis of Me_2Pd-tmeda, [L_nPd]$_2$-tmeda and L_nPd-DMSO

Me_2Pd-tmeda, **[L_nPd]$_2$-tmeda** and **L_nPd-DMSO and L_1Pd-NH_2PEG** pre-catalysts were synthesized according to previous literature report.[2,3]

Synthesis of L_1Pd-NR$_3$

In a 25 mL side-arm flask, **L_1Pd-DMSO** (400 mg, 0.67 mmol), N,N-dimethylhexylamine (1.2 equiv.) and dichloromethane (10 mL) were mixed. After stirring for an hour at room temperature, the solvent dichloromethane was removed under reduced pressure. The catalyst was rinsed three times with 4mL of diethyl ether and three times with 4mL of

pentane to remove the excess N,N-dimethylhexylamine. **L_1Pd-NR_3** was obtained as light pale yellow solid material (330mg, 76%).

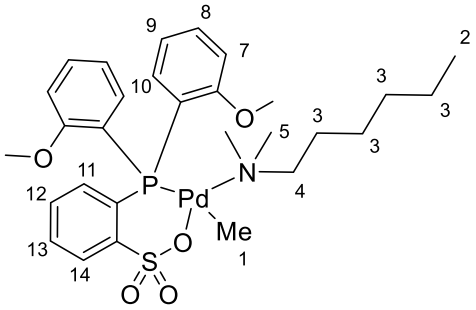

^{1}H NMR (500 MHz, CD_2Cl_2) δ= 8.0 (m, 1H, H-14), 7.59 (m br, 2H, H-8), 7.52 (m, 3H, H-10 and H-13), 7.44 (m, 1H, H-12), 7.26 (m, 1H, H-11), 7.01 (t, *J*=7.5, 2H, H-9), 6.94 (dd, *J*=4.6, 8.24, 2H, H-7), 3.6 (s, 6H, O*CH_3*, H-6), 2.6 (br. s, 8H, H-4,5), 1.3 (m, 8H, H-3), 0.9 (m, 3H, H-2), 0.0 (d, *J*=2.4, 3H, H-1). ^{31}P NMR (500 MHz, CD_2Cl_2): δ 22.5 Anal. Calcd for $C_{29}H_{40}N_1O_5S_1P_1Pd$: C 53.42%, H 6.18%, N 2.15% 57.96; Found: C 53.26%, H 6.06%, N 2.15%.

Ethylene Polymerization

All ethylene polymerizations were carried out in mechanically stirred (1000 rpm) a 100 mL stainless steel high-pressure reactor equipped with a heating and cooling jacket and thermocouple. The ethylene pressure was kept constant throughout the polymerizations. Before each polymerization run, the reactor was purged under vacuum at 85°C before to be backfilled with argon. Three more cycles of vacuum-and-backfilling with argon were repeated before the transfer of reagents in the reactor.

Ethylene polymerization in toluene

(Table 1, entries 1, 4 - 7 and 8, Supplementary Figures 4 - 9)

Stock solutions of pre-catalyst were prepared in dichloromethane or toluene. (1.8µmol/mL for **L_1Pd-NR$_3$** and 0.88µmol/mL for **L_2Pd-DMSO**)

Stock solutions of additives (N,N-dimethylhexylamine, water, THF, BCP and homopolymers) were prepared in toluene and added last in the reactor to study the influence of their concentration on the TO in Supplementary Figures 4 - 9. Water concentration were achieved by using a table of water solubility at different temperature.[4]

<u>100% Toluene</u>

100 mL toluene were transferred into the pre-heated (85°C) ethylene polymerization reactor. 0.5 mL of the pre-catalyst stock solution (0.9 µmol) was transferred into the reactor which was then pressurized to 40 bar. After the desire polymerization time ethylene was quickly vented. The polymer solution was precipitated into excess methanol and the polyethylene was collected by filtration. The polymer was dried under vacuum at 50°C overnight before performing any analysis.

<u>9:1 Toluene/water</u>

90 mL toluene were transferred into the pre-heated (85°C) ethylene polymerization reactor. 0.5mL of the pre-catalyst stock solution was transferred into the reactor followed by the addition of 10 mL water. The reactor was then pressurized to 40 atm. After the desire polymerization time ethylene was quickly vented. The polymer solution was precipitated into excess methanol and the polyethylene was collected by filtration. The polymer was dried under vacuum at 50°C overnight before performing any analysis.

Miniemulsion polymerizations

(Table 1, entry 2)

Ethylene polymerization in miniemulsion was performed using reported method.[3] The catalyst precursor was dissolved in 2 mL of dichloromethane and a 0.25 mL of hexadecane was added. This organic solution was added to SDS aqueous solution (20 mL of deionized water and 0.75 g SDS) and the resultant biphasic mixture was ultra-sonicated for 2 mins at 40% Amplitude using a: 505 Sonic Dismembrator using ¼” probe from Fisher Scientific. The resulting miniemulsion solution was transferred by cannula into the reactor preloaded with 80 mL water at temperature 85°C. The reactor was pressurized to achieve the desired pressure in the vessel and polymerization was run for 1 h. The reactor was then vented and brought to room temperature.

Specific weights of reaction solution were dried at 120°C overnight.

TO were calculated via gravimetric analysis using the weight difference of dry samples before and after ethylene polymerization.

Polymerization in water

(Table 1, entry 3)

Ethylene polymerization in water was performed using reported method.[3] 100mL of water were transfer into the pre-heated (85°C) ethylene polymerization reactor. The catalyst precursor and the surfactant was added to the reactor. The reactor was pressurized to achieve the desired pressure in the vessel and polymerization was run for 1 h. The reactor was then vented and brought to room temperature.

Specific weights of reaction solution were dried at 120°C overnight.

TO were calculated via gravimetric analysis using the weight difference of dry samples before and after ethylene polymerization.

Polymerization via Micellization

(Tables 2, 4 and 5, Figure 4)

A specific amount of BCP was dissolved in tetrahydrofuran (5 mL) into a 250 ml Schlenk flask before to add the pre-catalyst. Water (95 mL) was added dropwise to the above solution at 35-40°C over 15-20 min. This solution was filtered and transferred by cannula to the reactor at 85°C before to be pressurized at the desired pressure of ethylene and left to react the desired time.

Specific weights of reaction solution were dried at 120°C overnight.

TO were calculated via gravimetric analysis using the weight difference of dry samples before and after ethylene polymerization.

GPC sample preparation:

Portion (~15-30mL) of latexes solutions were dried overnight at 120°C. ~15mL of THF was added to the dry samples before to be placed in a shaker at 40°C overnight. THF was then carefully removed and the solid residue was washed with THF at room temperature. Finally the PE sample were dried overnight at 80°C before to be submitted for GPC.

Characterization of the Polyethylene

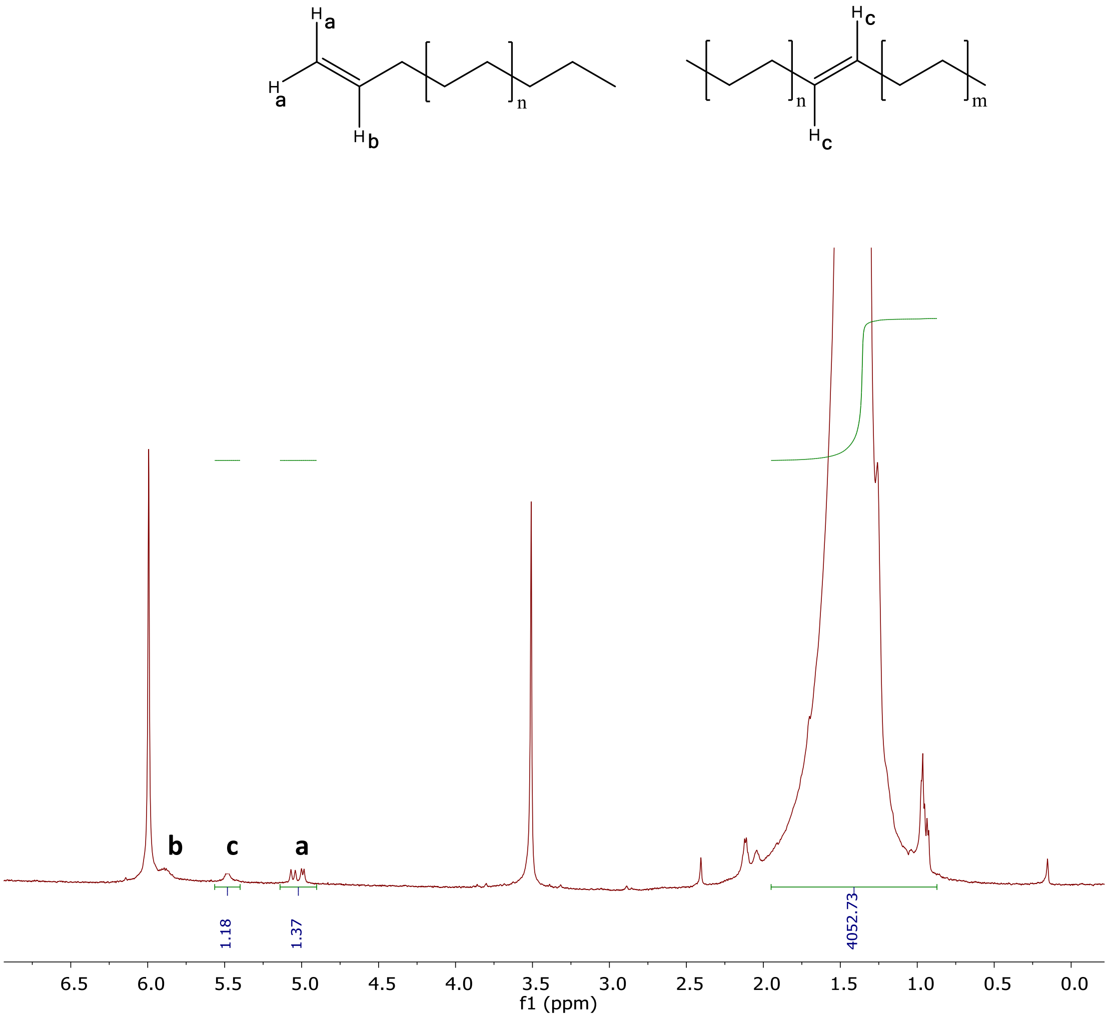

Supplementary Figure 2 Representative ^{1}H NMR (600 MHz, in $C_2D_4Cl_2$, at 120°C) of the polyethylene

Supplementary Note 1

TO h^{-1} as function of additives concentration in toluene

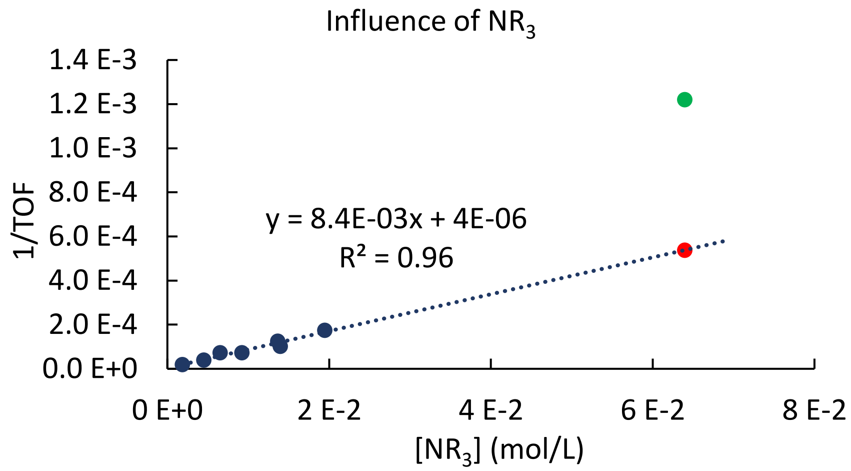

Supplementary Figure 3 Influence of N,N-dimethylhexylamine
Influence of the concentration in N,N-dimethylhexylamine ([NR_3]) on the catalyst activity (TOF) in toluene.

● Experimental data points

● Experimental 1/TOF measure with micelle (TOF = 820 h^{-1})

● Predicted 1/TOF at micelles concentration: 6.4 E-2 $mol.L^{-1}$ (TOF = 1860 $h-^{1}$)

Assumptions to find ● and ● : Micelles volume = 0.25mL, NR_3 fully dissociated of **2**, Ethylene solubility same in micelle and in toluene

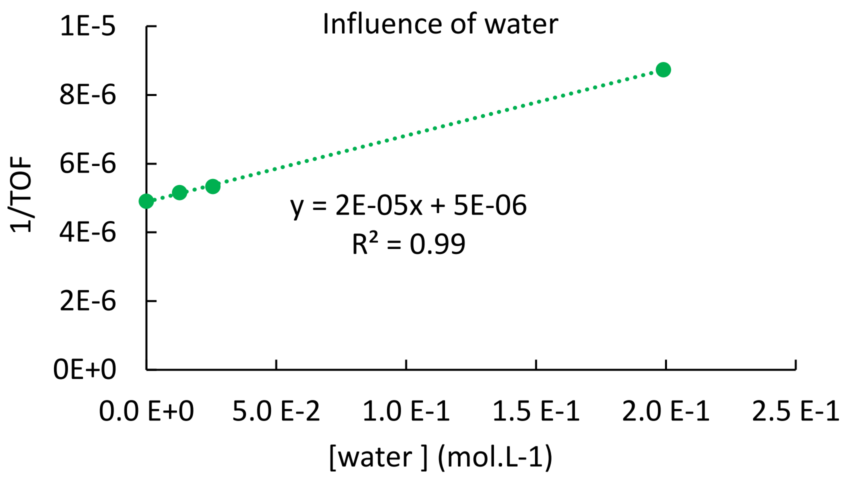

Supplementary Figure 4 Influence of water
Influence of the concentration in water ([water]) on the catalyst activity (TOF) in toluene.

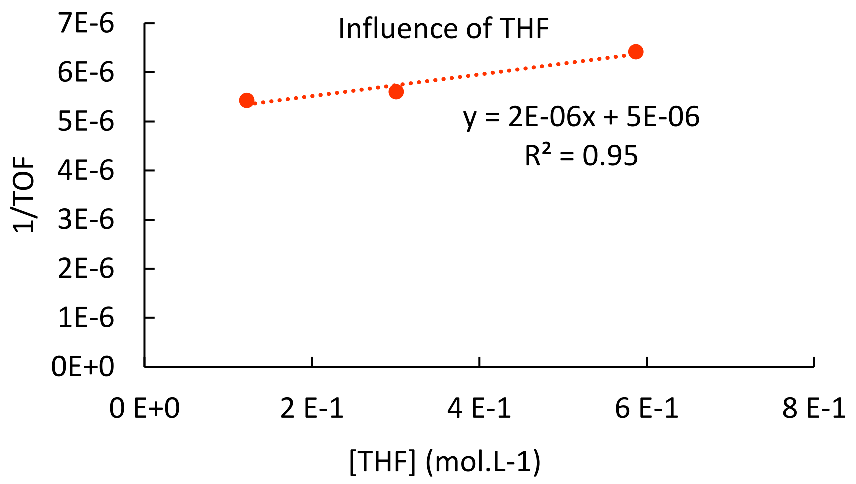

Supplementary Figure 5 Influence of THF
Influence of the concentration in THF ([THF]) on the catalyst activity (TOF) in toluene.

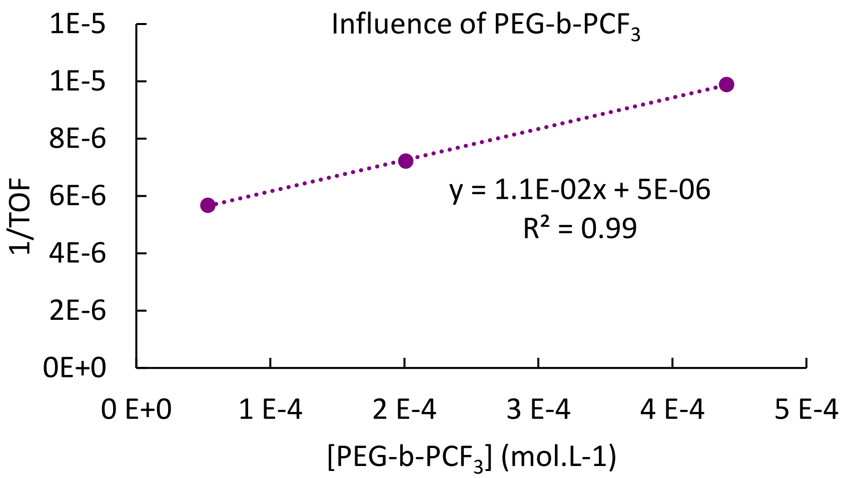

Supplementary Figure 6 Influence of PEG-*b*-PCF_3
Influence of the concentration in PEG-*b*-PCF_3 ([PEG-*b*-PCF_3]) on the catalyst activity (TOF) in toluene.

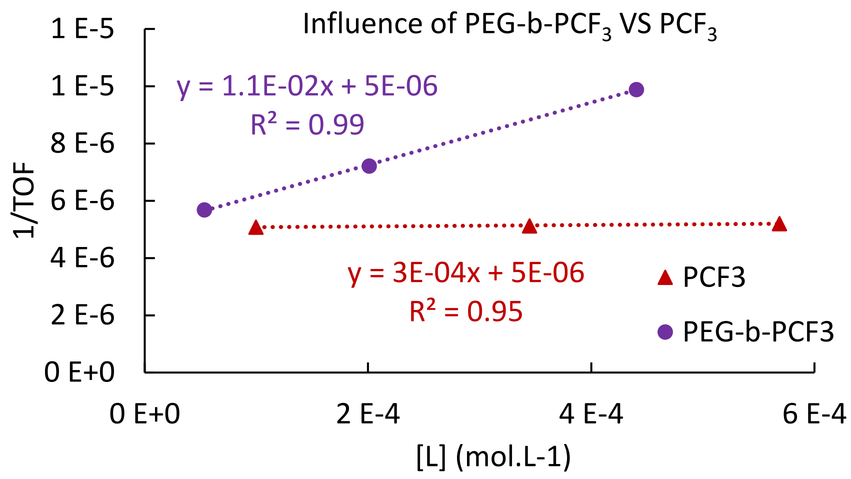

Supplementary Figure 7 Influence of PCF_3
Influence of the concentration of PCF_3 ([PCF_3]) on the catalyst activity (TOF) in toluene (▲) compared to the influence of [PEG-*b*-PCF_3] (●). L describe potential ligand here: PEG-*b*-PCF_3 and PCF_3.

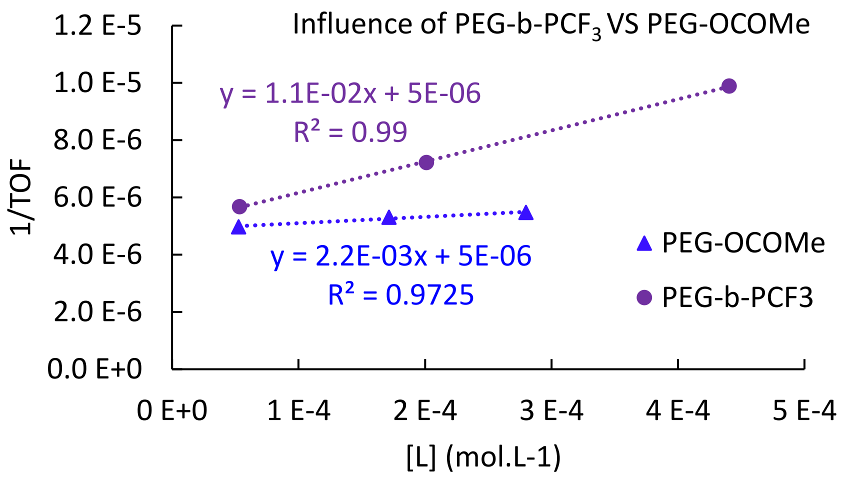

Supplementary Figure 8 Influence of PEG-OCOMe
Influence of the concentration in PEG-OCOMe ([PEG-OCOMe]) dependency on TOF in toluene (▲) compared to the influence of [PEG-b-PCF$_3$] (●).L describe potential ligand here: PEG-*b*-PCF$_3$ and PEG-OCOMe.

Equation derivation

$$K_{eq} = \frac{[2][L]}{[1][C_2H_4]}$$

$$TOF = \frac{R_p}{[Pd]_0} = \frac{R_p}{[1] + [2]}$$

$$\mathbf{R_p = k_p[2]}$$

$$\mathbf{R_p = K_{eq}k_p \frac{[1][C_2H_4]}{[L]}}$$

$$R_p = K_{eq}k_p([Pd]_o - [2])\frac{[C_2H_4]}{[L]}$$

$$R_p = K_{eq}k_p([Pd]_o - \frac{R_p}{k_p})\frac{[C_2H_4]}{[L]}$$

$$\frac{R_p}{K_{eq}k_p} * \frac{[L]}{[C_2H_4]} = [Pd]_o - \frac{R_p}{k_p}$$

$$R_p(\left(\frac{1}{K_{eq}k_p} * \frac{[L]}{[C_2H_4]} + \frac{1}{k_p}\right) = [Pd]_o$$

$$\frac{R_p}{k_p}\left(\frac{1}{K_{eq}} * \frac{[L]}{[C_2H_4]} + 1\right) = [Pd]_o$$

$$\frac{R_p}{[Pd]_o}\left(\frac{1}{K_{eq}} * \frac{[L]}{[C_2H_4]} + 1\right) = k_p$$

$$TOF = \frac{k_p}{\left(\frac{1}{K_{eq}} * \frac{[L]}{[C_2H_4]} + 1\right)}$$

$$\frac{1}{TOF} = \frac{1}{K_{eq} * k_p * [C_2H_4]}[L] + \frac{1}{k_p}$$

Micelles data

Supplementary Table 2 Average size of micelles with PEG-*b*-PCF_3

Entry	Micelles treatment	PS[a] [nm]	PDI
1	None	26	0.049
2	85°C for 1h	29	0.062
3	50%vol dilution[b]	29	0.110
4	With catalyst[c]	25	0.042

Stock micelles solution : 20 mg PEG-*b*-PCF_3 per mL of (water/THF, 95:5) solution a) Average particle size by volume determined by DLS, b) final BCP concentration 10mg/mL, c) L_1Pd-NR_3, 16µmol/100mL of (water/THF, 95:5) solution

DLS data

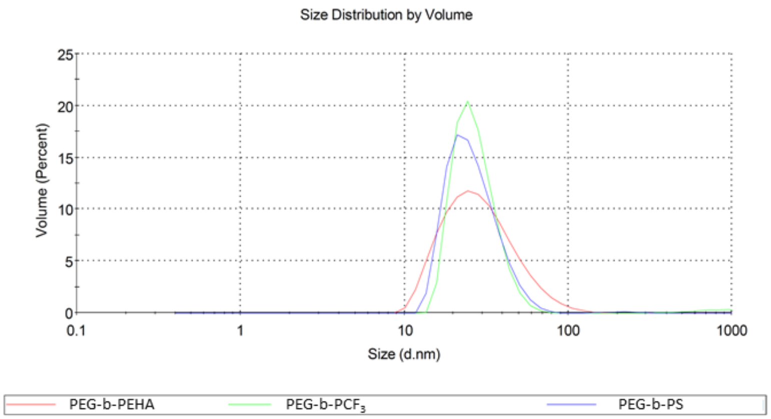

BCP	PS (nm)	Pdi
PEG-*b*-PEHA	31	0.308
PEG-*b*-PCF3	27	0.09
PEG-*b*-PS	27	0.135

Supplementary Figure 9 DLS of Micelles with PEG-*b*-PEHA, PEG-*b*-PCF$_3$ and PEG-*b*-PS

Supplementary Table 3 *DLS data Table 2:*

	Before polymerization		After polymerization	
	PS (nm)	Pdi	PS (nm)	Pdi
Table 2 Entry 1	24	0.032	117	0.366
Table 2 Entry 2	26	0.042	84	0.221
Table 2 Entry 3	26	0.079	57	0.143
Table 2 Entry 4	26	0.033	154	0.251
Table 2 Entry 5	25	0.096	68	0.099

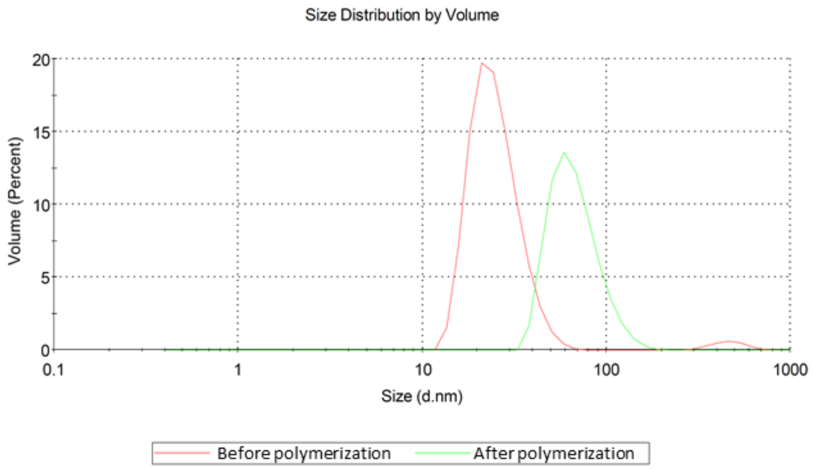

Supplementary Figure 10 Representative DLS of micelles with L_1Pd-NR_3 Before and after polymerization (table 2, entry 5)

Supplementary Table 4 *DLS data Table 4:*

	Before polymerization		After polymerization	
	PS (nm)	Pdi	PS (nm)	Pdi
Table 4 Entry 1	30	0.121	115	0.484
Table 4 Entry 2	28	0.041	163	0.189
Table 4 Entry 3	24	0.079	80	0.139
Table 4 Entry 4	27	0.135	67	0.130

Supplementary Table 5 *DLS data Table 5:*

	Before polymerization		After polymerization	
	PS (nm)	Pdi	PS (nm)	Pdi
Table 5 Entry 1	27	0.078	66	0.806
Table 5 Entry 2	26	0.095	112	0.218
Table 5 Entry 3	26	0.039	104	0.507
Table 5 Entry 4	27	0.086	103	0.515

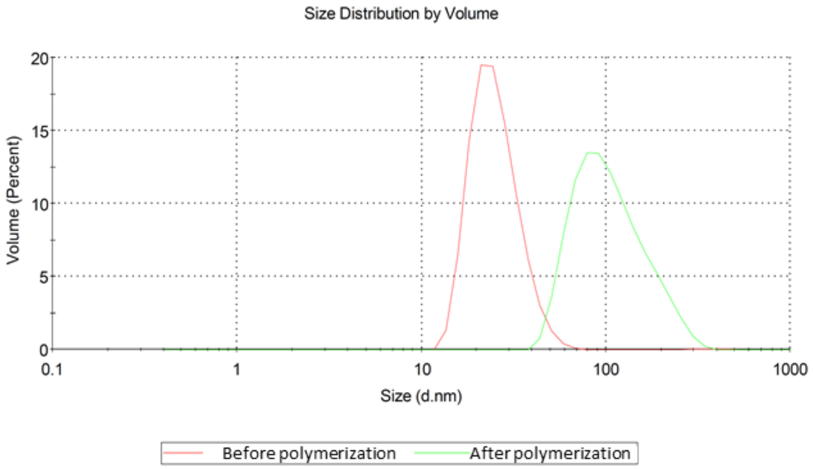

Supplementary Figure 11 Representative DLS of micelles with L_2Pd-DMSO Before and after polymerization (table 5, entry 2).

a

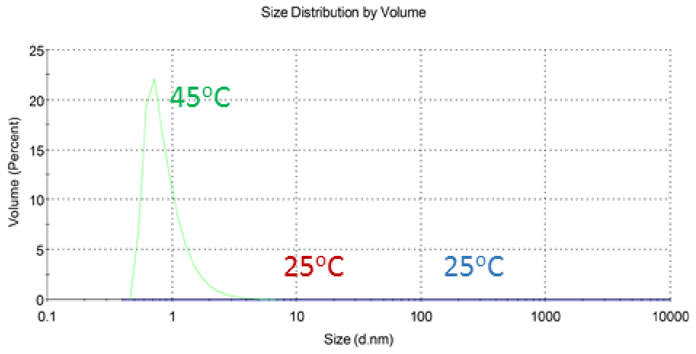

b

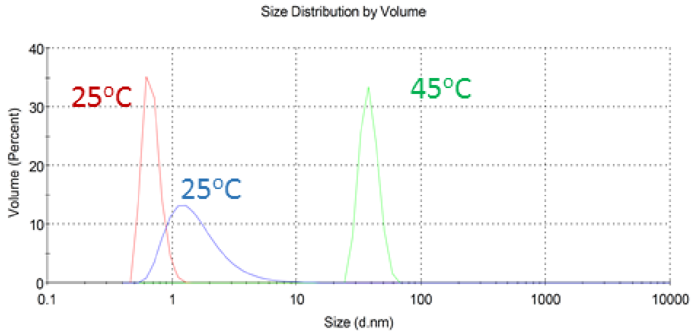

c

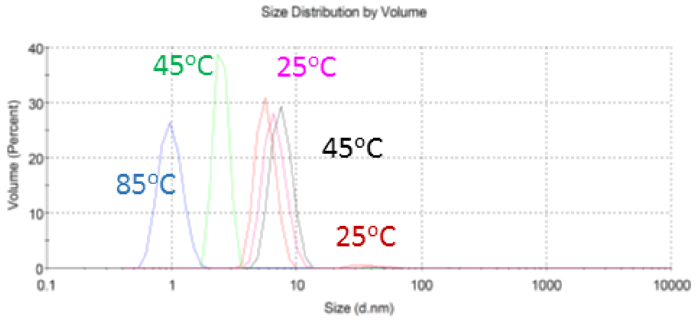

Supplementary Figure 12 DLS of homopolymers and block copolymer in toluene
a) DLS of PCF_3 homopolymer in toluene. The sample was heated following the pattern 25°C → 45°C →25°C. b) DLS of PEG-OCOMe homopolymer in toluene. The sample was heated following the pattern 25°C → 45°C →25°C. c) DLS of PEG-*b*-PCF_3 block copolymer in toluene The sample was heated following the pattern 25°C → 45°C →85°C → 45°C → 25°C.

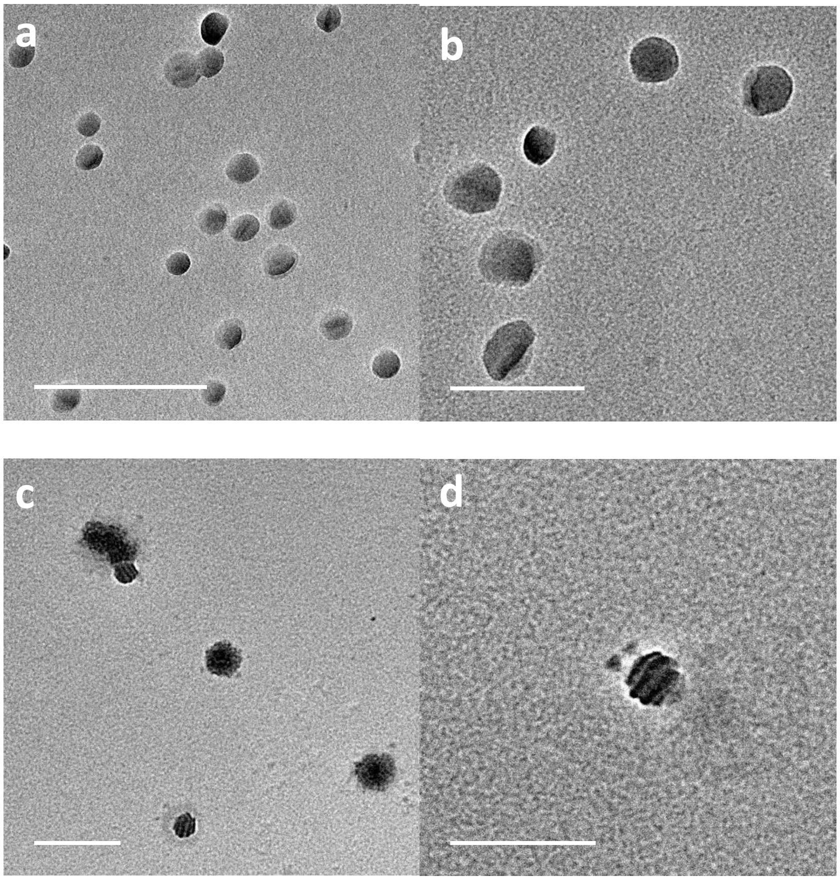

Supplementary Figure 13 Additional TEM images
Top: TEM of polymer particles obtained with L_1Pd-NR_3 (**table 2**, entry 2), Scale a) 500nnm, b) 200nm
Bottom: TEM of polymer particles obtained with L_2Pd-DMSO (**table 5**, entry 1), Scale c) 200nm, d) 100nm

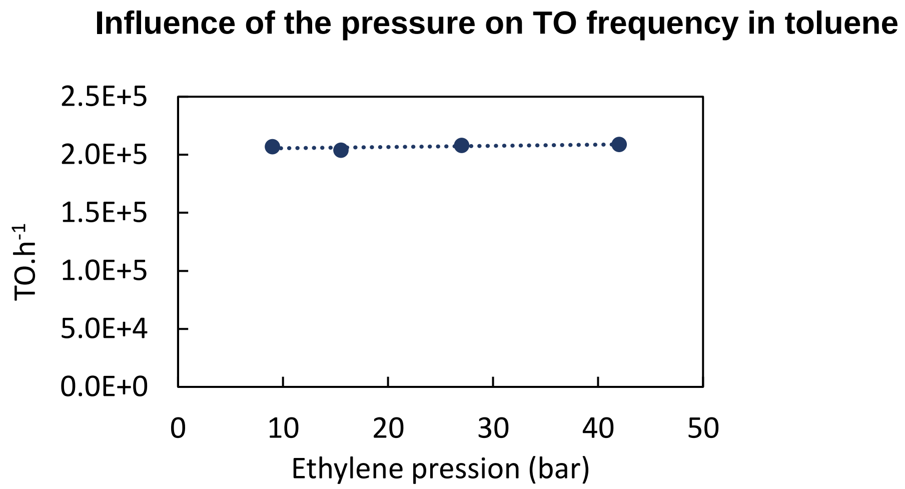

Supplementary Figure 14 [C_2H_4] dependency on TO frequency in toluene

Supplementary References

1. Skupov, K. M. *et al.* Palladium aryl sulfonate phosphine catalysts for the copolymerization of acrylates with ethene. *Macromol. Rapid Commun.* **28,** 2033–2038 (2007).

2. Guironnet, D., Roesle, P., Rünzi, T., Göttker-Schnetmann, I. & Mecking, S. Insertion Polymerization of Acrylate. *J. Am. Chem. Soc.* **131,** 422–423 (2009).

3. Zhang, D., Guironnet, D., Göttker-Schnetmann, I. & Mecking, S. Water-Soluble Complexes [(κ^2 - *P,O* -Phosphinesulfonato)PdMe(L)] and Their Catalytic Properties. *Organometallics* **28,** 4072–4078 (2009).

4. Hefter G.T. Quantive Solubility Studies of the Toluene - Water System. *NIST, IUPAC* **SDS-37,** 369 (1986).
